# Case Report: The Neuromusclar Triad of Immune Checkpoint Inhibitors: A Case Report of Myositis, Myocarditis, and Myasthenia Gravis Overlap Following Toripalimab Treatment

**DOI:** 10.3389/fcvm.2021.714460

**Published:** 2021-08-16

**Authors:** Yue-Bei Luo, Weiting Tang, Qiuming Zeng, Weiwei Duan, Shuyu Li, Xiaosu Yang, Fangfang Bi

**Affiliations:** Department of Neurology, Xiangya Hospital, Central South University, Changsha, China

**Keywords:** myositis, myasthenia gravis, myocarditis, PD-1, toripalimab

## Abstract

The neuromuscular adverse events of immune checkpoint inhibitor (ICI) treatment include myositis, polymyalgia rheumatica, myocarditis, and myasthenia syndrome. We report a 47-year old female presenting with external ophthalmoplegia, generalized muscle weakness, and third-degree atrioventricular block 4 weeks after toripalimab treatment for metastatic thymoma. Creatine kinase was elevated to 25,200 U/l and cardiac troponin I to 2.796 ng/ml. Autoantibody profiling shows positive anti-ryanodine receptor and anti-acetylcholine receptor antibodies and negative myositis specific antibodies. Repetitive nerve stimulation did not reveal decrement of compound muscle action potentials. Pulse methylprednisolone and immunoglobulin infusion, together with temporary pacemaker insertion normalized her muscle enzyme levels and cardiac rhythm. This is the first report of overlaping neuromuscular adverse event of toripalimab.

## Introduction

Immune checkpoint inhibitors (ICIs) such as antibodies targeting programmed cell death 1 (PD-1), PD-1 ligand (PD-L1), and cytotoxic T lymphocyte associated antigen 4 (CTLA-4) are the major advances in cancer therapy in the recent two decades. These drugs eliminate cancer cells by “releasing the brakes” on T cell activation pathway, i.e., enhancing immune surveillance. This modulatory mechanism inevitably leads to pleiotropic immune-related adverse events (irAEs) which include neuromuscular involvement manifesting as myositis, polymyalgia rheumatica, myocarditis myasthenia syndrome, and peripheral neuropathy.

Toripalimab (Junshi Bioscience) is a new PD-1 monoclonal antibody approved by the National Medical Products Administration of China in 2018. A phase I trial registered with U.S. National Library of Medicine (identifier NCT03474640) is underway in the United States. Here we describe a case presenting with polymyositis, myocarditis, and myasthenia gravis (MG) after toripalimab treatment.

## Case Description

**Figure 1:**
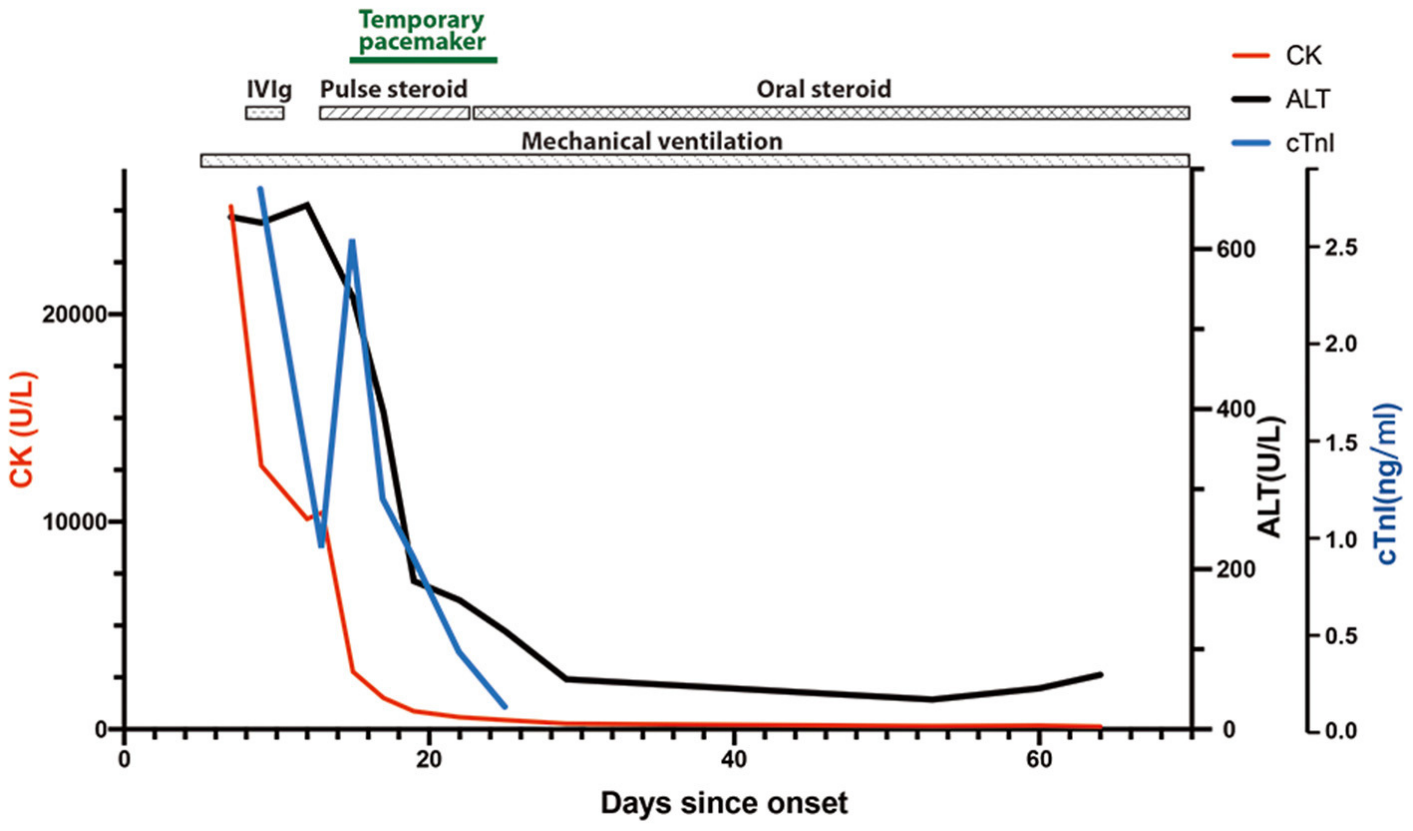
Illustration of the main laboratory markers and treatment of this patient.

A 47-year-old woman was admitted to the Emergency Department of Xiangya Hospital because of progressive diplopia, myalgia, and limb weakness for 7 days. The patient presented with non-fluctuating diplopia, myalgia, and weakness throughout the four limbs without prodromal infections. The symptoms rapidly deteriorated and she also developed dysphagia and dyspnea. She was treated with 240 mg toripalimab for bone metastasis of thymoma 4 weeks earlier. Type B2 thymoma was diagnosed 2 years ago, with immunohistochemistry showing CD5 (++), CD17 (–), TdT (++), p63 (+++), CK5/6 (+++), TTF (–), NapsinA (–), CR (–), and WT1 (–). She had no prior history of muscle weakness. On physical examination, she was alert and had a slurred speech. She also had bilateral ptosis, and her eyes were fixed in the mid-position without any noticeable horizontal or vertical movement. Pupillary light reflexes were normal. Muscle strength of proximal limbs was graded 3/5 and distal 3+/5. Deep tendon reflexes were absent. Cardiac troponin I level was increased to 2.796 ng/ml (normal range <0.04 ng/ml), and creatine kinase to 25,200 U/L (40–200 U/l). Electrocardiogram showed sinus tachycardia and right bundle branch block. Electromyography showed muscle unit potentials with reduced amplitude and short duration, as well as increased fibrillation and positive sharp weave in her biceps brachii, extensor digitorum communis and quadriceps femoris. Repetitive stimulation of the facial, accessory and ulnar nerves did not reveal significant decrement or increment. Intramuscular neostigmine of 1 mg did not improve her muscle weakness. The patient soon developed type II respiratory failure, therefore was incubated and mechanically ventilated. She was treated with intravenous immunoglobulin (0.4 g/kg/d for 5 days) and was later transferred to Neurology Intensive Care Unit. Subsequent screening for MG antibodies showed positive ryanodine receptor antibody (RyR-Ab) and acetylcholine receptor antibody (AChR-Ab, 7.11 nmol/l, normal range <0.45 nmol/l). Myositis specific and myositis related antibody profiling revealed weakly positive anti-fibrillarin, anti-NOR-90, and Ro-52 antibodies. A regime of pulse methylprednisolone of 500 mg, succeeded by 250 mg each for 5 days was initiated. On the same day, she developed third-degree atrioventricular block with multiple asystole events and temporal pacemaker was inserted. ECG monitoring showed her heart beat gradually returned to sinus rhythm and the pacemaker was removed 12 days later. After 10 days of pulse methylprednisolone, oral prednisolone (60 mg/d) was used for 4 weeks, then tapering to a dose of 50 mg/d. Repeated MG antibody test 40 days after disease onset demonstrated negative RyR-Ab, but still increased AChR-Ab level (9.66 nmol/l). Sixty-nine days after onset, distal limb strength of this patient improved to grade 4-/5 and proximal to 3/5. Despite the normalized CK level and increased muscle strength, she still had difficulty weaning from the ventilator. She was transferred to local hospital for pulmonary and extremity rehabilitation. Telephone follow-up 1 month after the referral indicated that she was off mechanical ventilator support and on non-invasive ventilation. Figure 1 shows chronological changes of main laboratory markers and treatment.

## Discussion

Neuromuscular irAEs represent a group of serious side effects of ICIs that requires prompt investigation and care. So far reported skeletal muscle irAEs of ICIs assume the forms of polymyositis, dermatomyositis, inclusion body myositis, antisynthetase syndrome, immune-mediated necrotizing myopathy, granulomatous myositis, and orbital myositis. The majority of ICI-related myositis cases assume the polymyositis phenotype, as our case does, although a definitive diagnosis requires pathological evidence. Patients usually present with acute onset myalgia and proximal weakness with moderately to severely elevated CK levels within 2 months of ICI initiation. Bulbar, extraocular muscle and axial involvement is also common. Myopathology ranges from necrotizing myopathy with few infiltrates to granulomatous inflammation within muscle tissues. Other types of myositis are anecdotal. ICI-related DM patients manifest characteristic DM skin changes such as periorbital heliotrope rashes. Whether the pathognomic perifascicular atrophy is present in this group of patients needs further exploration as unambiguous muscle biopsy evidence is lacking (1, 2). In a single case with lung adenocarcinoma, Nivolumab leads to development of myopathy and interstitial pneumonia, with anti-PL-7 seropositivity (3). Pembrolizumab is reported to cause painless orbital myositis with hyperCKaemia in a renal cancer case (4). In terms of the myositis specific antibodies that develop after ICI treatment, TIF1-γ and HMGCR antibodies are the only two reported antibodies (1, 5, 6). Notably, TIF1-γ antibody is closely correlated with tumors in idiopathic inflammatory myopathies. A definitive link between ICIs and HMGCR antibody remains undetermined, as the reported case has a pre-existing statin induced myopathy that resolves after statin discontinuation 2 weeks prior to ICI treatment. Patients with pre-existing myositis are inclined to flares following ICI treatment (7, 8). Proposed mechanisms include loss of self-tolerance by disrupted balance between Treg and T effector cells, shared antigens between tumor and tissue, and epitope spreading in which release of tumor-origin and self antigens initiates inflammatory cascade (9, 10).

Admittedly, our patient does not show any response to neostigmine and her repetitive stimulation lacks the characteristic amplitude decrement. It is also estimated that 20–37% of thymoma patients with elevated AChR levels do not develop MG (11, 12), which challenges the diagnosis of MG in this case. However, neither myositis nor myocarditis could account for the early and severe involvement of extraocular and respiratory muscles of this patient. Previous studies estimate that approximately 0.12–0.4% patients receiving ICI treatment develop or experience flares of MG (13, 14). The characteristic amplitude decrement of compound muscle action potentials occurs in 29–53% of these patients. Up to 66% of ICI-related MG cases have elevated anti-AChR antibody titer (13). They have more frequent anti-striated muscle antibodies than the idiopathic cases (66.7 vs. 39.5%) (13, 15). This group of patients also have a higher propensity to develop respiratory failure and a worse outcome. Anti-RyR antibody positive MG patients typically present with ocular, bulbar and axial weakness instead of limb weakness. Our case falls into class V of MG according to MGFA classification. This patient displays a generalized involvement of virtually all modalities of striated muscles, including extraocular, facial, bulbar, respiratory, axial, limb and cardiac muscles. While it is difficult to dissect the specific roles of each of the neuromuscular triad in phenotype development, there is no doubt that they synergistically contribute to the aggressive disease progression.

Adverse cardiac events related to ICIs include myocarditis, pericardial diseases, myocardial infarction, and vasculitis. The severity of myocarditis cases following ICI therapy range from sub-clinical to fatal. Patients may present with dyspnea, palpitation, syncope, or chest pain. Despite the overall elevated troponin levels, approximately half of cases demonstrate abnormal left ventricular ejection fraction (16, 17). In comparison, 89% cases have arrhythmias including atrial fibrillation, premature ventricular contraction, conduction block and ventricular tachycardia. On cardiovascular magnetic resonance, 48% of patients show late gadolinium enhancement (LGE), whose patterns include transmural, sub-epicardial, mid-myocardial, and diffuse (17). LGE was present in anteroseptal, inferoseptal, inferior, and inferolateral wall. Endomyocardial biopsy findings are characterized by myocardial infiltration consisting of T lymphocytes and macrophages (18). Notably, a previous analysis of irAEs in Chinese patients shows that toripalimab causes myocarditis more frequently than other ICIs (19). The National Comprehensive Cancer Network recommends permanent discontinuation of ICIs in Grade 3 (severe) and 4 (life-threatening) myocarditis cases (20).

Overall, the myositis-myocarditis-myasthenia gravis overlap represents a serious neuromuscular irAE of PD-1 monoantibodies, and are considerably more deleterious compared with their idiopathic counterparts. Meta-analysis shows that myositis, myocarditis and myasthenia gravis account for one fifth of irAE-related death (21). When the neuromuscular triad is present, the disease progression is particularly malignant. Permanent discontinuation of ICIs is therefore recommended for severe myositis or myocarditis, and also for myositis-myocarditis overlap (22, 23). Patients require more aggressive treatments including pulse steroid, immunoglobulin infusion and plasmapheresis. A second immunomodulatory drug is in option if the patient shows poor response to the above therapies (24).

It should be noted that thymic epithelial tumors (TETs) are associated with higher incidence of ICI-related irAEs compared with other types of cancers (25, 26). This may be explained by the critical role of thymus in T lymphocyte education and the compromised immune tolerance of TETs. Toripalimb is a recombinant humanized IgG4 monoantibody against PD-1 molecule that has a promising therapeutic potential for advance solid tumors. It is reported to cause more aggressive myocarditis (grade 3–5) (19). In conclusion, we report a case presenting with the neuromuscular triad irAE after toripalimab treatment. Clinicians should carefully consider the type and nature of tumors and be aware of the potential irAEs in each ICIs when planning immunotherapy.

## Data Availability Statement

The original contributions presented in the study are included in the article/Supplementary Material, further inquiries can be directed to the corresponding author/s.

## Ethics Statement

The studies involving human participants were reviewed and approved by the Ethic Committee of the Xiangya Hospital of Central South University. The patients/participants provided their written informed consent to participate in this study.

## Author Contributions

Y-BL and FB initiated the study. Y-BL and WT collected clinical data and wrote the manuscript. FB and XY reviewed the manuscript. QZ, WD, WT, SL, XY, and FB provided medical care for the patient and participated in literature reviewing. All authors contributed to the article and approved the submitted version.

## Conflict of Interest

The authors declare that the research was conducted in the absence of any commercial or financial relationships that could be construed as a potential conflict of interest.

## Publisher's Note

All claims expressed in this article are solely those of the authors and do not necessarily represent those of their affiliated organizations, or those of the publisher, the editors and the reviewers. Any product that may be evaluated in this article, or claim that may be made by its manufacturer, is not guaranteed or endorsed by the publisher.
